# AI-based pulmonary artery to ascending aorta ratio on non-contrast CT for pulmonary hypertension: diameter vs. volume assessment

**DOI:** 10.1093/ehjimp/qyag146

**Published:** 2026-08-24

**Authors:** Turki Nasser Alnasser, Alireza Hokmabadi, Ahmed Maiter, Michael Sharkey, Christopher Johns, Smitha Rajaram, David G Kiely, Samer Alabed, Andrew J Swift

**Affiliations:** School of Medicine and Population Health, The University of Sheffield, Sheffield S10 2TN, UK; Radiological Sciences Department, College of Applied Medical Science, King Saud bin Abdulaziz University for Health Science, Riyadh 11426, Saudi Arabia; King Abdullah International Medical Research Centre (KAIMRC), Riyadh 11426, Saudi Arabia; School of Medicine and Population Health, The University of Sheffield, Sheffield S10 2TN, UK; Insigneo Institute, Faculty of Engineering, The University of Sheffield, Sheffield, UK; School of Medicine and Population Health, The University of Sheffield, Sheffield S10 2TN, UK; Insigneo Institute, Faculty of Engineering, The University of Sheffield, Sheffield, UK; Department of Clinical Radiology, Sheffield Teaching Hospitals, Sheffield, UK; National Institute for Health and Care Research, Sheffield Biomedical Research Centre, Sheffield, UK; School of Medicine and Population Health, The University of Sheffield, Sheffield S10 2TN, UK; Department of Clinical Radiology, Sheffield Teaching Hospitals, Sheffield, UK; Department of Clinical Radiology, Sheffield Teaching Hospitals, Sheffield, UK; School of Medicine and Population Health, The University of Sheffield, Sheffield S10 2TN, UK; Insigneo Institute, Faculty of Engineering, The University of Sheffield, Sheffield, UK; National Institute for Health and Care Research, Sheffield Biomedical Research Centre, Sheffield, UK; Sheffield Pulmonary Vascular Disease Unit, Sheffield Teaching Hospitals NHS Trust, Sheffield, UK; School of Medicine and Population Health, The University of Sheffield, Sheffield S10 2TN, UK; Insigneo Institute, Faculty of Engineering, The University of Sheffield, Sheffield, UK; Department of Clinical Radiology, Sheffield Teaching Hospitals, Sheffield, UK; National Institute for Health and Care Research, Sheffield Biomedical Research Centre, Sheffield, UK; School of Medicine and Population Health, The University of Sheffield, Sheffield S10 2TN, UK; Insigneo Institute, Faculty of Engineering, The University of Sheffield, Sheffield, UK; Department of Clinical Radiology, Sheffield Teaching Hospitals, Sheffield, UK; National Institute for Health and Care Research, Sheffield Biomedical Research Centre, Sheffield, UK

**Keywords:** pulmonary hypertension, CT, diagnostic, pulmonary artery, ascending aorta, volume, diameter

## Abstract

**Aims:**

To assess the diagnostic accuracy of a deep learning (DL) model for quantifying the pulmonary artery (PA) and ascending aorta (AAo) diameters and volumes on non-contrast computed tomography (CT) scans for detecting pulmonary hypertension (PH).

**Methods and results:**

The PA and AAo were segmented using a validated DL model on non-contrast CT scans. PA/AAo ratios were quantified using diameter and volume measurements. Testing was performed using two independent patient cohorts. A first cohort (*n* = 97) was used to compare DL-derived and manual diameter-based PA/AAo ratios and to evaluate their diagnostic accuracy against right heart catheterization (RHC). A second cohort (*n* = 425; 385 internal and 40 external patients) was then used to compare the diagnostic performance of the DL-derived diameter- and volume-based PA/AAo ratios using RHC as the reference standard. In the first cohort, 97 patients were included (mean age 63 ± 13 years, 50% female, and 62 patients with PH). Agreement with the diameter-based approach was good [Observer 1: intraclass correlation coefficient (ICC) = 0.79 (0.70–0.85); Observer 2: ICC = 0.79 (0.70–0.86)]. Agreement with volume-based PA/AAo ratio was moderate [Observer 1: ICC = 0.56 (0.41–0.68); Observer 2: ICC = 0.55 (0.39–0.67)]. The DL diameter-based (area under the curve (AUC) = 0.83) and volume-based (AUC = 0.84) PA/AAo ratio outperformed the manual measurements by Observer 1 (AUC=0.72) and Observer 2 (AUC = 0.74). The second cohort comprised 425 patients (385 internal and 40 external), with a mean age of 65 ± 12 years, 60% female, and 353 patients with PH. The volume-based approach achieved superior diagnostic accuracy compared with the diameter-based approach (AUC 0.85 vs. 0.73), with higher sensitivity (66% vs. 41%) and specificity (89% vs. 82%).

**Conclusion:**

The PA/AAo volume ratio on non-contrast CT demonstrates high diagnostic accuracy for detecting PH and could assist with opportunistic detection of PH in patients undergoing routine chest imaging.

## Graphical Abstract

**Graphical Abstract:**
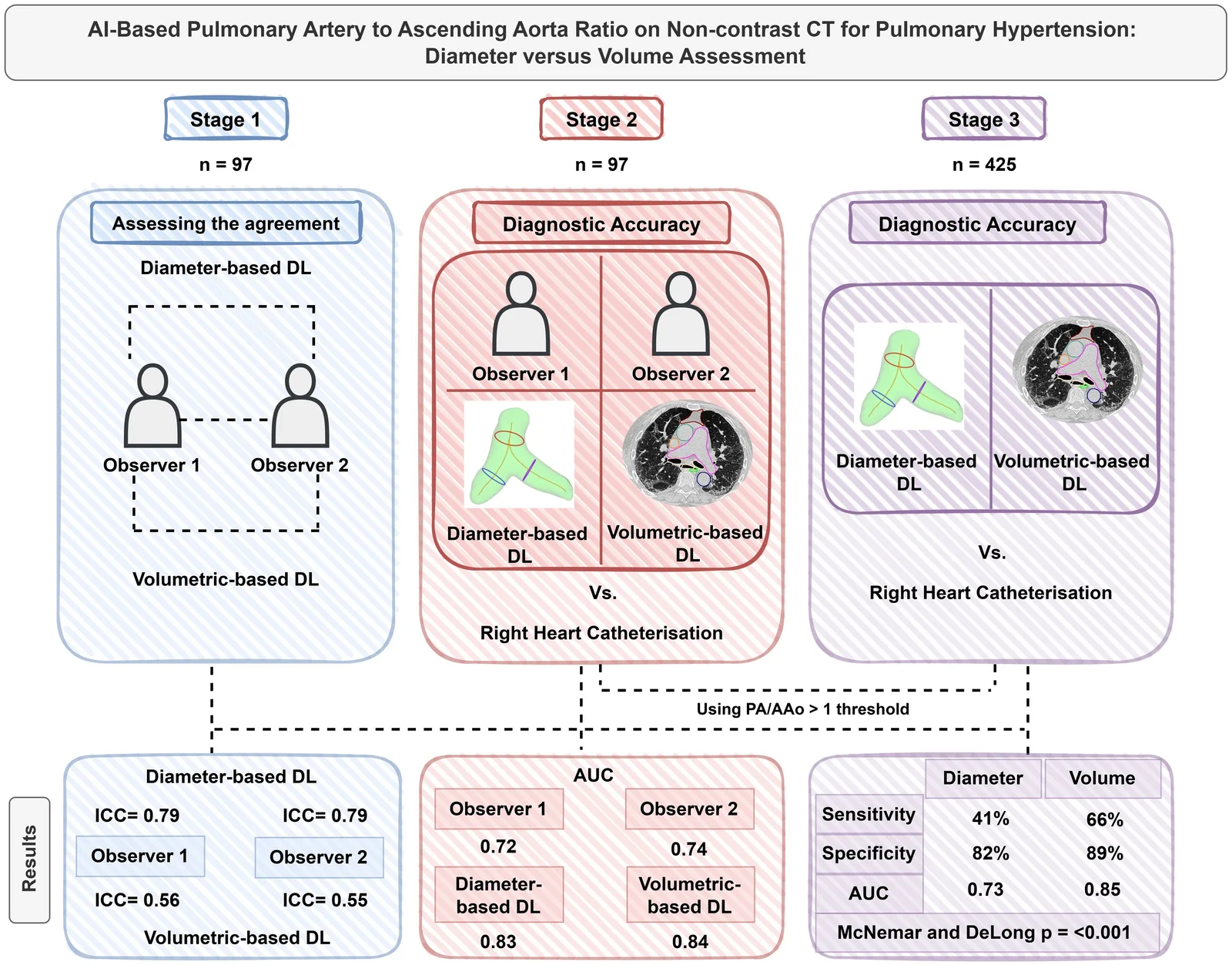

## Introduction

Pulmonary hypertension (PH) is characterized by elevated pulmonary artery pressure. Left unchecked, this causes progressive right heart dysfunction that results in substantial morbidity and mortality.^1,2^ PH has a number of causes and is classified into five groups: pulmonary arterial hypertension (PAH, Group 1), PH due to left heart disease (Group 2), lung disease and/or hypoxia (Group 3), chronic thromboembolic pulmonary hypertension (Group 4), and PH with unclear or multifactorial mechanisms (Group 5). All PH, regardless of the underlying cause, is defined haemodynamically by a mean pulmonary artery pressure (mPAP) >20 mmHg when measured invasively on right heart catheterization (RHC).^1^

Although RHC is the gold standard for diagnosing PH and assessing the degree of haemodynamic disturbance in the disease, it is an invasive investigation that is resource-intensive and carries potentially significant procedure-related risks.^3,4^ Given the invasive nature of RHC, there is a clear clinical need for reliable, non-invasive methods to support the evaluation of PH, both to guide appropriate patient selection for invasive testing and to facilitate earlier detection in symptomatic individuals, as well as opportunistic screening in at-risk or asymptomatic populations.^5^ Non-contrast computed tomography (CT) is widely available and is recommended in international guidelines to assess lung parenchyma in suspected PAH or PH secondary to lung disease.^1^

Chronic elevation of pulmonary artery pressure causes dilatation of the main pulmonary artery. This is an important structural hallmark of PH that can be identified on CT.^1,6^ A pulmonary artery to ascending aorta diameter ratio (PA/AAo) >1 on CT is associated with an mPAP >20 mmHg on RHC and highly predicts PAH.^6,7^

While the current literature has focused primarily on manual diameter-based PA/AAo measurements for the diagnosis of PH,^6,7^ these manual approaches are time-consuming and susceptible to human error and inter-observer variability. Deep learning (DL), by contrast, has demonstrated accuracy, robustness, and efficiency in the segmentation and quantification of cardiac and vascular structures on CT scans^8–10^ and can provide anatomical measurements, including volumetric metrics in addition to diameter-based measurements.^8–13^

This study aimed to evaluate the diagnostic accuracy of the PA/AAo ratio on non-contrast CT, using two-dimensional diameter-based and three-dimensional volumetric-based measurements obtained from DL-derived vascular segmentations, with invasive RHC haemodynamics as the reference standard in internal and external cohorts. A secondary aim was to assess agreement between DL-derived PA/AAo ratios and manually measured PA/AAo diameter ratios obtained by experienced observers.

## Methods

### Ethical approval

This study received ethical approval from an Institutional Review Board (Ethics Committee Name: East of England - Cambridge East Research Ethics C, Approval Code: REC 22/EE/0011, Approval Date: 24 February 2022). The Assessing the Spectrum of Pulmonary Hypertension Identified at a Referral Centre (ASPIRE) registry is an ethically approved research database managed by Sheffield Teaching Hospitals NHS Foundation Trust (Reference: STH14169, REC 22/EE/0011). In accordance with UK regulations, written consent was not required for the analysis of routinely collected de-identified clinical data collated as part of an ethically approved research database (National guidance within the Integrated Research Application System).

### Study population and included CT scans

Eligible patients and their CT scans were identified retrospectively from the ASPIRE registry. The registry comprises consecutive patients who have undergone investigation for suspected PH at the Sheffield Pulmonary Vascular Disease Unit (Sheffield, UK) without having received any prior PH treatment. Patients within the registry have typically undergone specialist clinical review, lung function testing, multimodal imaging, and RHC.^14^ Patients within the ASPIRE registry were eligible for inclusion if they were aged ≥18 years, had a suspected diagnosis of PH, and had undergone investigation with both RHC and non-electrocardiogram-gated non-contrast CT in a 30-day period. Scans were included if the slice thickness was 5 mm or less with a field of view of 400 × 400 mm and an acquisition matrix of 512 × 512. Slice thickness was not further standardized across cases, and all analyses were performed using the original image spacing values. Restricting inclusion to scans with slice thicknesses of 5 mm or less ensured sufficient through-plane resolution for reliable three-dimensional reconstruction and quantitative analysis, including vessel diameter and volume measurements. Patients were excluded if they did not undergo both RHC and non-contrast CT within the specified timeframe. The CT scans were acquired using different multidetector scanners (GE MEDICAL SYSTEMS, Siemens, and Canon) with standard acquisition helical mode parameters: pitch ∼1, tube current 100–500 mA, and 120 kV.

### Study flow and data splitting

**Figure 1:**
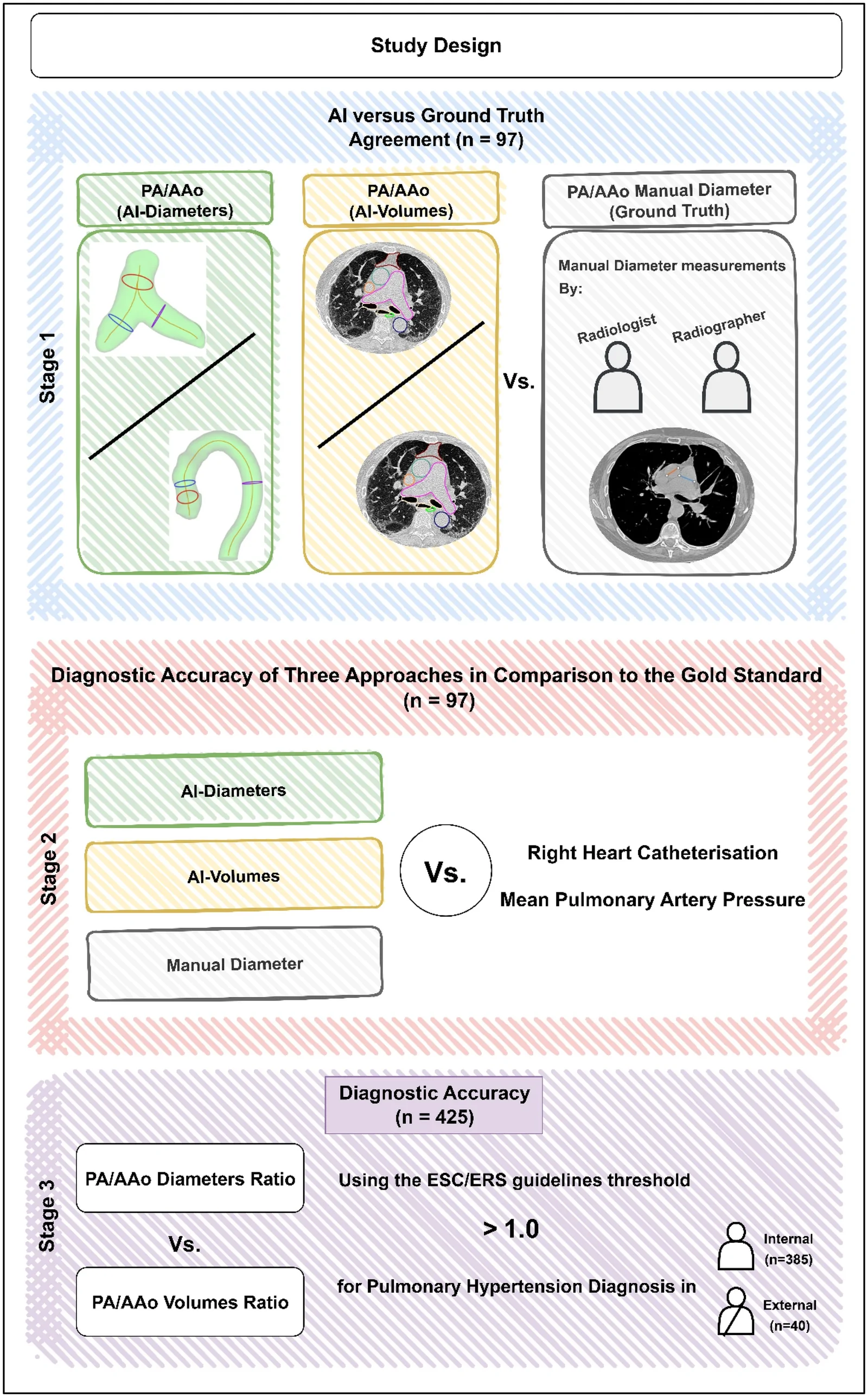
Flow chart of the study design, including AI measurement agreement with the reference standard, three approaches to diagnostic accuracy, and AI diagnostic accuracy stage.

The flow of the study is summarized in *Figure 1*. Two datasets of non-contrast chest CTs performed between 2007 and 2018 were randomly selected from the registry. This study was conducted in three stages. First, a cohort of 97 non-contrast CT scans was used to compare diameter- and volume-DL-based PA/AAo ratios with the manual PA/AAo diameter ratio, assessing inter-observer agreement (Stage 1) and diagnostic accuracy (Stage 2) for identifying PH, with RHC as the reference standard. PA/AAo ratios were used for all analyses, thereby enabling direct comparison between manual diameter-based and DL-derived diameter- and volume-based measurements independent of measurement units. In the final stage, 425 patients (385 internal and 40 external) were included to compare the diagnostic accuracy of the DL-based diameter and volumetric PA/AAo ratios for the diagnosis of PH with RHC as the reference standard. The validated threshold of a PA/AAo ratio >1 was used as indicative of PH throughout the stages.^1,5^ Although the threshold has been historically validated for diameter-based PA/AAo assessment, the same threshold was applied to volumetric ratios to permit direct comparison between approaches.

### Manual measurements of PA/AAo diameter

The manual measurements of PA and AAo diameters were conducted in 97 scans by a cardiothoracic radiologist with 13 years of experience (AJS) and a cross-sectional imaging radiographer with 6 years of experience (TNA) using 3D Slicer (version 5.6.2).^15^ The manual measurements were checked and confirmed by a cardiothoracic radiologist with 7 years of experience (SA).

### Segmentation model development and post-processing

The DL segmentation model used in this study was developed and validated in earlier work.^16^ In brief, the model was built using the nnU-Net framework,^17^ and this established model formed the basis for extracting the PA and AAo measurements used in the current analysis.

### Diameter-based measurement pipeline

**Figure 2:**
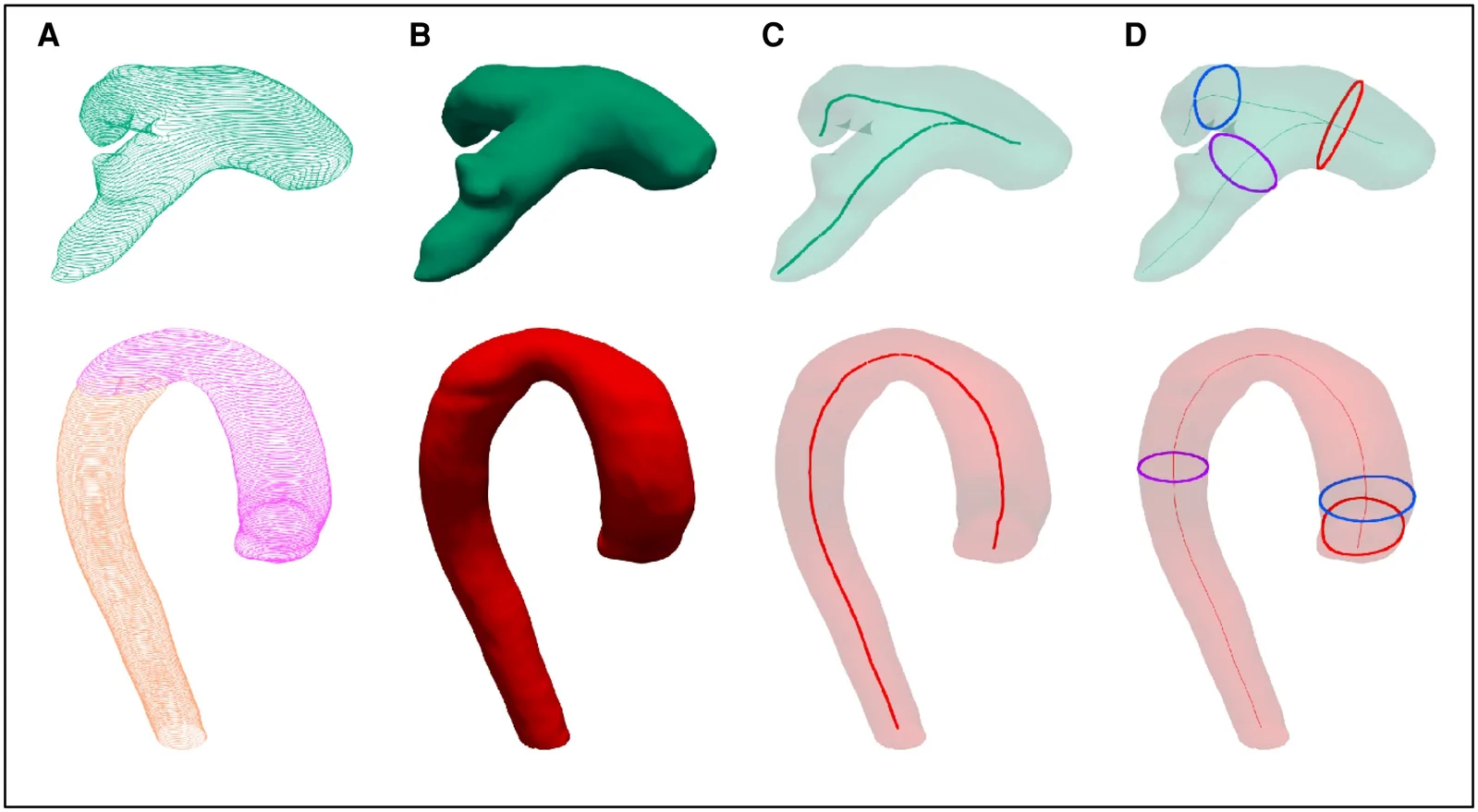
Diameter-based measurement pipeline for the pulmonary artery (top) and aorta (bottom). (*A*) Vessel contours from segmentation masks. (*B*) Three-dimensional surface meshes. (*C*) Vessel centrelines. (*D*) Automatically identified anatomical landmarks.

The segmentation model output included 11 anatomical masks. For this study, only the AAo, descending aorta (DAo), and PA were used. The AAo and DAo were then merged into a single aortic mask, yielding two final labels: aorta and pulmonary artery. Both were converted to 3D surface meshes in real-world coordinates using the marching-cubes implementation in the Vascular Modelling Toolkit (VMTK),^18^ an open-source library widely used for vascular modelling and centreline extraction. To improve surface continuity and reduce stair-step artefacts between slices, the binary segmentation masks were first transformed into signed distance maps and lightly smoothed using a Gaussian filter prior to mesh extraction. The resulting smoothed representations enabled more anatomically continuous vascular surfaces while preserving the underlying vessel geometry. A schematic overview of the complete diameter-based measurement workflow is shown in *Figure 2*.

After mesh generation, the centrelines of the aorta and PA were extracted using VMTK’s centreline algorithms. Once centrelines were obtained, cross-sectional diameters were computed at each centreline point. Automatic identification of anatomical landmarks was then performed. For the PA, the bifurcation was localized by identifying the region where the main trunk begins to diverge into two branches. Because bifurcation geometry varies among patients, an adaptive spatial threshold was used to identify a bifurcation zone rather than a single point. Within each branch, a stable measurement location was chosen by assessing local diameter uniformity and the spatial separation of the branches. Diameters for the main PA, right PA, and left PA were then computed by averaging the values within a small neighbourhood around each landmark while excluding outliers.

Aortic landmarks were determined using one of two strategies, depending on whether a reliable PA bifurcation plane could be identified:

1. Using the PA bifurcation as the anatomical reference, two axial planes were defined at 30 and 10 mm inferior to the bifurcation plane, corresponding approximately to the sinotubular junction (STJ) and mid-ascending aorta (mid-AAo), respectively.
2. When the PA bifurcation cannot be reliably identified (e.g. in cases where PA segmentation is incomplete or unavailable), an alternative reference strategy is used based on the z-axis extent of the AAo. This approach serves as a predefined fallback mechanism to ensure applicability in datasets with limited PA visibility. The mid-z position of the AAo segmentation served as an internal reference plane, from which surrogate planes were placed 20 and 40 mm superiorly to approximate the STJ and mid-AAo levels, respectively.

**Figure 3:**
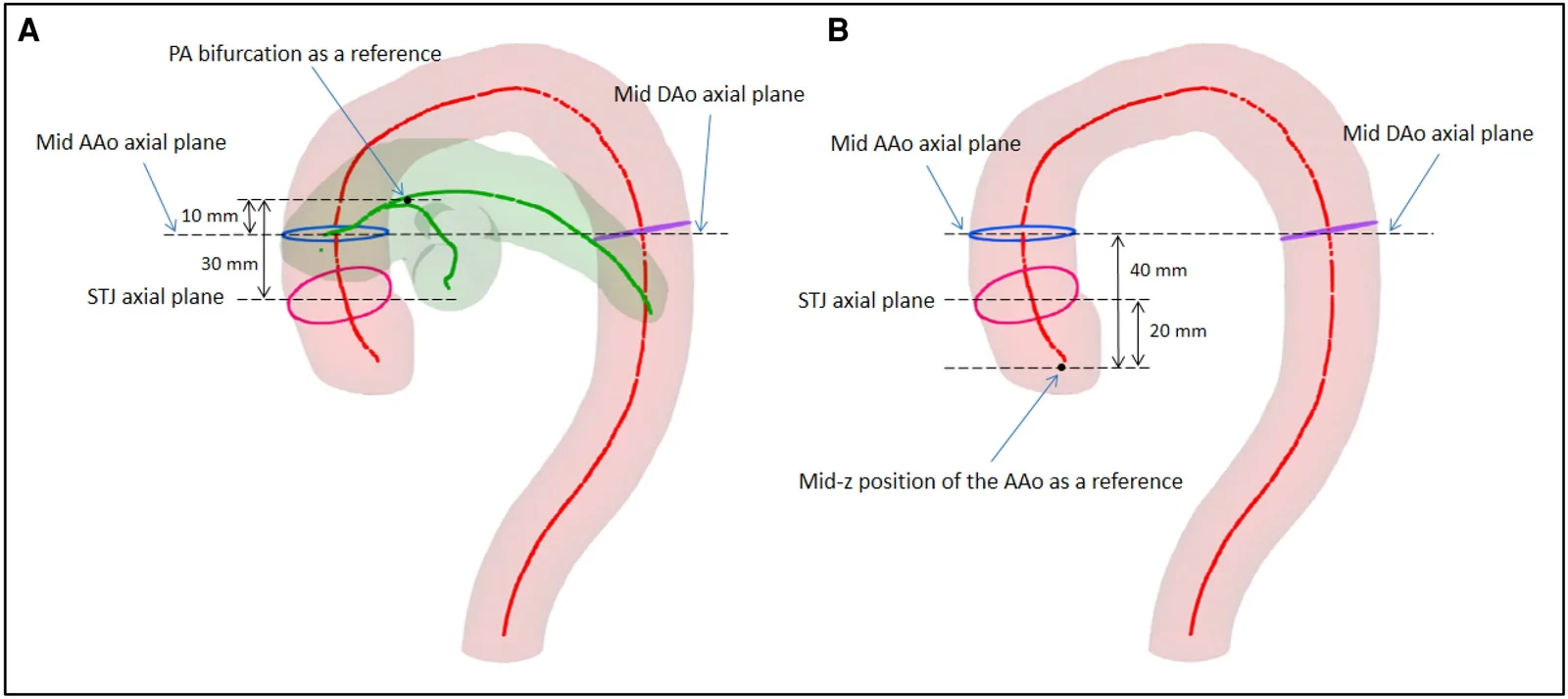
Schematic illustration of the two aortic landmarking strategies: (*A*) primary strategy using the PA bifurcation as the anatomical reference; (*B*) secondary strategy based solely on AAo geometry.

In both approaches, vessel diameters were calculated as the mean of cross-sectional measurements within a local neighbourhood, while excluding clear outliers to minimize the effects of segmentation inaccuracies or mesh artefacts. A schematic overview of both landmarking strategies is provided in *Figure 3*.

### Volume-based measurements

Volumes for the PA and AAo were computed directly from their binary masks without any additional resampling or reorientation. For each structure, the volume was obtained by summing all labelled voxels and multiplying by the respective voxel volume. In this study, the measured PA volume included the segmented PA region as defined by the model output, covering the main PA and proximal branches included within the segmentation mask, while the aortic volume corresponded to the segmented AAo only. Given the consistent in-plane resolution and adequate z-axis coverage across all scans, these volumetric measurements were feasible and directly comparable across subjects.

### Statistical analysis

Continuous variables are presented as mean ± standard deviation (SD). Using a threshold of PA/AAo >1, sensitivity, specificity, positive predictive value (PPV), and negative predictive value (NPV) were calculated from 2 × 2 contingency tables and are presented with 95% confidence intervals (CIs). Receiver operating characteristic analysis was performed with calculation of area under the curve (AUC) values. Paired comparisons between the two DL approaches were performed using McNemar’s test for binary classification and DeLong’s test for differences in AUC. Agreement between the observers and the DL-derived results was measured using intra-class correlation (ICC) and 95% CI. Agreement was further assessed using Bland–Altman analysis. Systematic bias was calculated as the mean difference between measurements, and 95% limits of agreement were defined as mean difference ± 1.96 SD of the differences. As the absolute values of volumes (in millilitres) and diameters (in millimetres) are not directly comparable, the PA/AAo ratio was calculated for each approach, and these ratio values were compared. The statistical significance threshold α was defined as 0.05. All statistical analyses were performed using SPSS Statistics for Windows (Version 29.0; IBM Corp, 2022), except for DeLong’s test, which was conducted in R (version 4.5.1).

## Results

### Patient characteristics

The demographics of the included patients are summarized in *Table 1*. Ninety-seven patients were included in the cohort used for Stage 1 and Stage 2 (50% female, mean age 63 ± 13 years, 64% with confirmed PH, mean mPAP 38.9 ± 17 mmHg). Four hundred twenty-five patients were included in the cohort for Stage 3. Three hundred eight-five underwent their CT scans in Sheffield (60% female, mean age 65 ± 12 years, 82% with confirmed PH, mean mPAP 41.5 ± 13.8 mmHg). Forty underwent their CT scans in 23 different centres (58% female, mean age 65 ± 12 years, 90% with confirmed PH, mean mPAP 41 ± 12.4 mmHg). A total of 82% of patients were White British according to documented self-reported ethnicity. Among patients with Group 1 PAH, only one had congenital heart disease–associated PAH, while the remaining cases predominantly comprised connective tissue disease–associated PAH (70%) and idiopathic PAH (27%). CT scans were performed using systems by GE Healthcare (91%), Canon (7%), and Siemens (2%). Summary values for PA diameter, AAo diameter, and PA/AAo ratio according to each measurement method are provided in the Supplementary material (Supplementary data online, *Table S1*).

**Table 1:** Demographic information of the patient population

		Stage 1 and 2 cohort (*n* = 97)	Stage 3 cohort (*n* = 425)	
		Agreement Diagnostic accuracy	Diagnostic accuracy	
		Internal cohort (*n* = 97)	Internal cohort (*n* = 385)	External cohort (*n* = 40)
**Age (years** **±** **SD)**		63 ± 13	65 ± 12	65 ± 12
**Gender**	Male	48 (49.5%)	153 (40.0%)	17 (42.5%)
	Female	49 (50.5%)	232 (60.0%)	23 (57.5%)
**Ethnicity**	White	80 (82.5%)	312 (81.0%)	34 (85.0%)
	Non-White	17 (17.5%)	46 (12.0%)	4 (10.0%)
	Not stated	0 (0%)	27 (7.0%)	2 (5.0%)
**Scanner manufacturers**	GE LightSpeed Pro 32	46 (47.4%)	162 (42.0%)	16 (40.0%)
	GE LightSpeed VCT	51 (52.6%)	184 (48.0%)	17 (42.5%)
	Siemens Definition AS+	0 (0%)	3 (0.7%)	2 (5.0%)
	Siemens Sensation	0 (0%)	3 (0.7%)	1 (2.5%)
	Canon Aquilion ONE	0 (0%)	33 (8.6%)	4 (10.0%)
**Scan dates**		2007–2018	2007–2018	2007–2018
**CT image slices (mean** **±** **SD)**		276 ± 83	140 ± 134	102 ± 112
**Diagnosis**	Group 1: PAH	13 (13.4%)	105 (27.3%)	8 (20.0%)
	Group 2: PH-LHD	18 (18.6%)	81 (21.0%)	8 (20.0%)
	Group 3: PH-Lung disease	18 (18.6%)	94 (24.4%)	13 (32.5%)
	Group 4: CTEPH	13 (13.4%)	30 (7.8%)	4 (10.0%)
	Group 5: Unclear PH	0 (0%)	7 (1.8%)	3 (7.5%)
	No PH	35 (36.0%)	68 (17.7%)	4 (10.0%)
**Pulmonary artery haemodynamics**	mPAP mean ± SD (mmHg)	38.9 ± 17.0	41.5 ± 13.8	41.0 ± 12.4
	PVR mean ± SD (dynes·sec·cm^−5^)	509.4 ± 447.2	546.2 ± 407.6	482.3 ± 346.1
	PAWP mean ± SD (mmHg)	12.0 ± 6.5	13.2 ± 6.0	13.9 ± 5.0

PAH, pulmonary arterial hypertension; PH, pulmonary hypertension; LHD, left heart disease; CTEPH, chronic thromboembolic pulmonary hypertension; mPAP, mean pulmonary arterial pressure; PVR, pulmonary vascular resistance; PAWP, pulmonary artery wedge pressure.

### Stage 1: agreement with manual measurements (*n* = 97)

Inter-observer agreement between Observer 1 and Observer 2 for manual diameter-based PA/AAo measurements was excellent (ICC = 0.94, 95% CI 0.91–0.96). Strong agreement was observed between PA/AAo ratios obtained from diameter-based DL and manual measurements. Diameter-based DL approach estimates demonstrated good agreement with both manual measurements (ICC = 0.79, 95% CI 0.70–0.85 for Observer 1; ICC = 0.79, 95% CI 0.70–0.86 for Observer 2). The volumetric-based DL PA/AAo showed moderate agreement with manual PA/AAo, with lower ICC values for both observers (ICC = 0.56, 95% CI 0.41–0.68 for Observer 1; ICC = 0.55, 95% CI 0.39–0.67 for Observer 2) (*Table 2*). Diameter-based DL demonstrated minimal bias and good agreement with both observers, whereas volumetric-based DL showed wider limits of agreement (see Supplementary data online, *Table S2*).

**Table 2:** Intra-class correlation with *P*-values assessing the agreement between DL models and manual measurements of the PA/AAO ratio (*n* = 97)

PA/AAo measurements correlation		Manual measurements	
		Observer 1	Observer 2
**DL measurements**	**Diameters**	ICC = 0.79 [0.70–0.85] *P* < 0.001	ICC = 0.79 [0.70–0.86] *P* < 0.001
	**Volumes**	ICC = 0.56 [0.41–0.68] *P* < 0.001	ICC = 0.55 [0.39–0.67] *P* < 0.001
**Observer 1 and Observer 2**		ICC = 0.94 [0.91–0.96], *P* < 0.001	

PA, pulmonary artery; AAo, ascending aorta; DL, deep learning; ICC, intra-class correlation.

### Stage 2: diagnostic accuracy of manual vs. DL-based PA/AAo ratio with RHC as the gold standard (*n* = 97)

**Figure 4:**
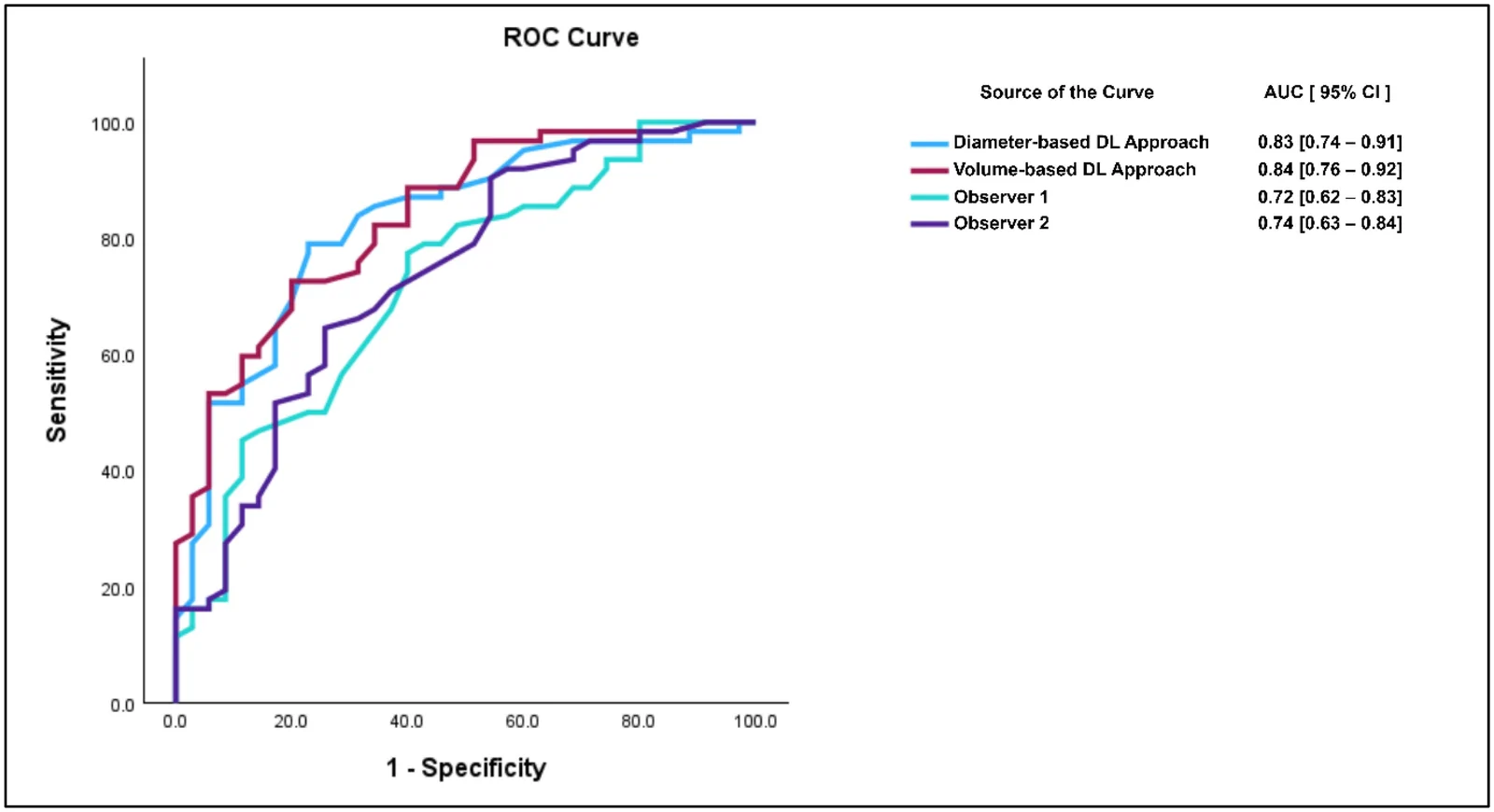
ROC curves illustrate the diagnostic accuracy of deep learning approaches and manual PA/AAo measurements in identifying pulmonary hypertension patients, validated against RHC (*n* = 97).

When benchmarked against RHC, both manual and DL-based PA/AAo approaches demonstrated moderate diagnostic utility, although the DL approaches consistently outperformed manual assessment. Manual measurements yielded sensitivities of 57% and 65% for Observers 1 and 2, with specificities of 71% and 74% and corresponding AUCs of 0.72 (95% CI 0.62–0.83) and 0.74 (95% CI 0.63–0.84), respectively. In comparison, the diameter-based DL PA/AAo maintained similar sensitivity (57%) while achieving a higher specificity of 86% and an AUC of 0.83 (95% CI 0.74–0.91). The volumetric-based PA/AAo ratio further improved the diagnostic accuracy, with a sensitivity of 60%, a specificity of 89%, and an AUC of 0.84 (95% CI 0.76–0.92) (*Table 3* and *Figure 4*). McNemar’s tests showed that neither DL-based approach differed significantly from Observer 1 (*P* = 0.383 for the diameter-based and *P* = 0.541 for the volumetric-based). Compared with Observer 2, the diameter-based showed a significant difference (*P* = 0.035), while the volumetric-based did not (*P* = 0.096). DeLong tests indicated that the AUCs of both DL-based measurements were significantly higher than those of Observer 1 (*P* = 0.012 and 0.009) and Observer 2 (*P* = 0.038 and 0.031) (see Supplementary data online, *Table S3*).

**Table 3:** Diagnostic performance metrics for DL-derived diameter, DL-derived volume, and manual diameter measurements compared with RHC (*n* = 97)

PA/AAo measurements		RHC (mPAP > 20 mmHg)				
		Sensitivity [CI]	Specificity [CI]	PPV [CI]	NPV [CI]	AUC [CI]
**Manual**	**Observer 1**	57% [43.3–69.0%]	71% [53.7–85.4%]	78% [62.9–88.8%]	48% [34.0–62.4%]	0.72 [0.62–0.83]
	**Observer 2**	65% [51.3–76.3%]	74% [56.7–87.5%]	82% [68.0–91.2%]	54% [39.2–68.6%]	0.74 [0.63–0.84]
**DL**	**Diameter**	57% [43.3–69.0%]	86% [69.7–95.2%]	88% [73.2–95.8%]	53% [39.0–66.0%]	0.83 [0.74–0.91]
	**Volume**	60% [46.4–71.9%]	89% [73.3–96.8%]	90% [76.9–97.3%]	55% [41.5–68.7%]	0.84 [0.76–0.92]

PA, pulmonary artery; AAo, ascending aorta; DL, deep learning; RHC, right heart catheterization; mPAP, mean pulmonary artery pressure; PPV, positive predictive value; NPV, negative predictive value; AUC, area under the curve; CI, confidence interval.

### Stage 3: diagnostic accuracy of diameter vs. volumetric DL-based PA/AAo with RHC as reference standard (*n* = 425, 385 internal and 40 external)

**Figure 5:**
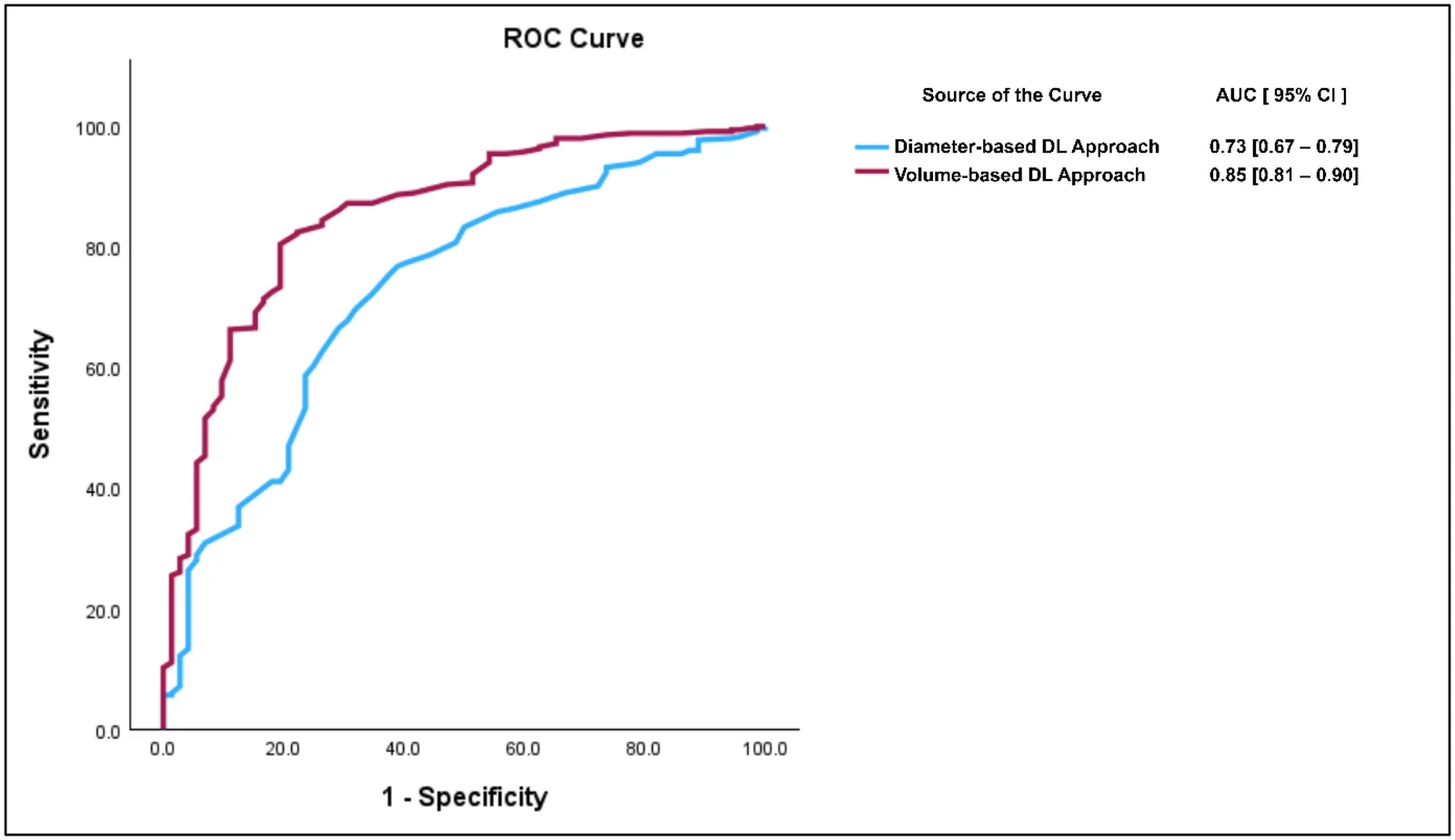
ROC curves illustrate the diagnostic accuracy of deep learning approaches of PA/AAO measurements in identifying pulmonary hypertension patients, validated against RHC (*n* = 425).

Volumetric DL-derived PA/AAo measurements (AUC = 0.85; 95% CI 0.81–0.90) had a higher diagnostic accuracy than diameter-based assessment (AUC = 0.73; 95% CI 0.67–0.79) when compared against RHC (*P* < 0.001). The volumetric-based PA/AAo ratio achieved both higher sensitivity (66%) and specificity (89%), while the diameter-based DL approach showed low sensitivity (41%) but maintained moderate specificity (82%). Both approaches demonstrated high positive predictive values of 92% and 97% for diameter- and volume-based PA/AAo ratios, respectively. However, negative predictive values remained comparatively low at 22% and 35% for diameter- and volume-based ratios, respectively. McNemar’s test indicated a statistically significant difference in classification between the two DL approaches (χ^2^ = 88.97, *P* < 0.001) (*Table 4* and *Figure 5*). The diagnostic accuracy of the diameter- and volumetric-based DL PA/AAo on the independent internal and external cohorts is presented in Supplementary data online, *Table S4*.

**Table 4:** Diagnostic performance metrics for DL-derived diameter and DL-derived volume compared with RHC (*n* = 425) (385 internal and 40 external)

	Diameter-based DL	Volumetric-based DL
**Sensitivity [CI]**	41% [36.5–46.0%]	66% [61.4–71.2%]
**Specificity [CI]**	82% [72.0–90.3%]	89% [81.6–96.2%]
**PPV [CI]**	92% [86.7–95.4%]	97% [94.5–98.9%]
**NPV [CI]**	22% [17.3–27.3%]	35% [28.1–41.9%]
**AUC [CI]**	0.73 [0.67–0.79]	0.85 [0.81–0.90]
**McNemar χ^2^ (*P*-value)**	88.97 (<0.001)	
**DeLong *P*-value**	<0.001	

DL, deep learning; PPV, positive predictive value; NPV, negative predictive value; AUC, area under the curve; CI, confidence interval.

### Comparison of aortic landmark selection approaches

In this study cohort, the primary landmark strategy was successfully applied in all cases, and no cases required activation of the fallback method. Nevertheless, to permit direct comparison between approaches, aortic measurements were recomputed using the secondary landmark strategy across the entire test dataset. Comparative analyses, including agreement statistics and downstream diagnostic performance metrics, are presented in the Supplementary data online, *Tables S5* and *S6*.

## Discussion

A PA/AAo ratio >1 is associated with an mPAP >20 mmHg and a diagnosis of PH. This study evaluated the accuracy of a DL-based pipeline for the measurement of the PA/AAo ratio on non-electrocardiogram-gated and non-contrast CT scans. Segmentations of the PA and aorta were obtained from non-contrast CT scans using an existing DL tool and processed in a novel pipeline to obtain diameter-based and volume-based PA/AAo ratio measurements. Agreement between the DL-derived measurements and manual measurements was assessed. Dichotomized DL-derived measurements were compared with dichotomized mPAP results from RHC to assess diagnostic accuracy for PH. As expected, the diameter-based DL approach showed the closest agreement with manual expert measurements, as both rely on similar two-dimensional diameter estimation. In contrast, the volumetric-based DL approach showed only moderate correlation with the manual reference standard, reflecting the inherent differences between three-dimensional segmentation-derived measurements and traditional axial slice-based techniques. However, volumetric analysis demonstrated higher diagnostic accuracy for PH, suggesting that this approach may better capture true vascular dilatation and elevated mPAP.

Although a range of DL architectures has demonstrated strong performance in CT segmentation,^8–10,19^ a previously developed DL segmentation model based on the nnU-Net framework^16^ was employed. The model was further refined through task-specific adaptation of preprocessing, resampling, and training procedures to optimize the performance.^17,19^ The segmentation model used for the analyses in this study demonstrated strong performance on internal and external cohorts.^16^ Specifically, it achieved a Dice similarity coefficient (DSC) of 0.90 and an HD95 of 1.6 mm for PA segmentation and a DSC of 0.94 with an HD95 of 0.9 mm for the AAo. These results are comparable to those reported for state-of-the-art frameworks such as TotalSegmentator.^20^ In the present work, further steps involved applying two post-processing strategies to quantify the PA/AAo ratio using both a diameter-based method that replicated standard clinical measurements and a volumetric-based method designed to exploit the full 3D segmentation output.

While prior studies have established the predictive value of the PA/AAo ratio using diameter-based metrics,^1,6,7,21^ our findings advance the field by providing robust evidence that DL-derived volumetric measurements on non-electrocardiogram-gated, non-contrast CT afford greater diagnostic discriminative power. The lower diagnostic performance of the diameter-based DL approach observed in Stage 3 compared with Stage 2 may reflect the substantially larger and more heterogeneous cohort used for Stage 3 evaluation, which included both internal and external datasets acquired across multiple centres and scanner platforms. The prevalence of PH differed between cohorts. The lower proportion of non-PH patients in Stage 3 likely reduced the distinction between normal and abnormal vascular morphology and may have negatively affected the discriminative performance of diameter-based measurements. Moreover, diameter-based measurements rely on localized two-dimensional assessment and are more susceptible to variability related to slice selection and anatomical landmark localization. Variability in CT acquisition parameters, particularly slice thickness, may have had an impact on diameter-based measurements in the larger and more heterogeneous Stage 3 cohort. Consistent with this, the Stage 3 cohort demonstrated greater variability in the number of CT slices per scan (140 ± 134 in Stage 3 vs. 276 ± 83 in Stage 2). Notably, this aligns with previous work demonstrating that volumetric measurements obtained from DL segmentation correlate strongly with haemodynamic measurements on RHC.^10^ A key strength of this study is that the model was evaluated in a substantial internal and external cohort comprising 425 patients with invasive haemodynamic data obtained from RHC. A consistent observation across all approaches was the high PPV accompanied by a comparatively low NPV. This pattern is likely attributable to the high prevalence of PH within our study population (i.e. 80%).^22,23^ Another possible factor is the biological heterogeneity in PA remodelling, particularly among patients with Group 2 and Group 3, in whom PA enlargement is less pronounced.^24,25^

The use of a volumetric-based DL approach to quantify the PA/AAo ratio on non-electrocardiogram-gated, non-contrast CT offers clinical advantages in the evaluation of suspected PH. In routine practice, PA and AAo diameters are typically measured manually by radiologists, a process that is inherently time-consuming and subject to inter- and intra-observer variability. These measurements are also sensitive to slice selection and cardiac motion, limitations that are accentuated in non-electrocardiogram-gated acquisitions.^9,10,26,27^ Although volumetric measurements remain susceptible to cardiac motion, they exploit the full three-dimensional anatomy of the vessels, providing a more comprehensive and physiologically representative assessment of PA enlargement.^28^ This study has shown that the combination of an accurate DL segmentation model with a volumetric approach to post-processing can yield automated measurements of the PA/AAo ratio on CT that are rapid, reproducible, robust, and of diagnostic value. Collectively, these advantages position volumetric assessment as a clinically valuable tool that may enhance diagnostic utility for PH assessment using widely available non-contrast CT scans as well as facilitate opportunistic screening on routine CT scans.^10,16^

This study has several limitations. First, the manual diameter reference standard measurements were obtained from only two experts from one institution. Secondly, the sample size was not determined by an *a priori* power calculation. The majority of the study population was White, and most scans were acquired on GE HealthCare scanners, which may limit the generalizability of the findings to other populations. The study was retrospective, and the impact of the pipeline on clinical practice was not assessed. McNemar and DeLong tests were not performed for comparisons between the internal and external cohorts, as the cohorts are independent and the external cohort was relatively small compared with the internal cohort. Furthermore, the study included only one patient with congenital heart disease (CHD)-associated PAH, limiting the ability to evaluate the diagnostic performance of the PA/AAo ratio across different PAH subtypes or to compare pressure-loaded and volume-loaded mechanisms of PA dilatation. Therefore, the diagnostic performance observed in this study may not be directly generalizable to patients with CHD, in whom PA dilatation may occur in the absence of PH. Future work should focus on multicentre studies with broader demographics and evaluating the impact of the pipeline on clinical practice to ensure reliability and generalizability. With regard to the low NPV observed across all approaches, future studies are encouraged to use a more balanced cohort to confirm the generalizability of these results.

## Conclusion

A novel post-processing pipeline was established to derive diameter-based and volume-based measurements of the PA/AAo ratio from the outputs of a DL segmentation tool on non-electrocardiogram-gated, non-contrast CT scans. As expected, the diameter-based approach showed better agreement with manual diameter-based reference standard measurements. However, the volumetric approach provided substantially better metrics of diagnostic accuracy for PH when compared with mPAP results from RHC. These findings indicate that reliable and diagnostically valuable volume-based measurements of PA/AAo can be derived automatically from non-electrocardiogram-gated, non-contrast CT scans. Although further work is required, this could provide a method for opportunistic screening of PH on routine chest CT scans.

## Supplementary Material

qyag146_Supplementary_Data

## Data Availability

De-identified data within the ASPIRE registry can be made available for analysis to researchers who provide a methodologically sound proposal that fits within the aims of the ASPIRE registry. The ASPIRE data management committee will assess each proposal and decide within 3 months after submission. A data access agreement signed by the lead researcher and a data sharing agreement between Sheffield Teaching Hospitals and the recipient institution will be required before data are shared. Information and application forms can be found at https://bit.ly/aspire-registry. The trained model and data underlying this article will be shared upon reasonable request for research purposes by non-commercial entities. Requests should be directed to the corresponding author.

## Lead author biography

Turki Alnasser is a researcher based in the UK and Saudi Arabia, with a focus on cardiac and thoracic CT imaging, specializing in medical imaging, artificial intelligence, deep learning, and medical image analysis.

He is currently completing his PhD at the University of Sheffield, UK, and works as a faculty member at King Saud bin Abdulaziz University for Health Sciences, Saudi Arabia.

## References

[1] Humbert M, Kovacs G, Hoeper MM, Badagliacca R, Berger RMF, Brida M et al 2022 ESC/ERS Guidelines for the diagnosis and treatment of pulmonary hypertension. Eur Heart J 2022;43:3618–731. 36017548 10.1093/eurheartj/ehac237

[2] Dwivedi K, Sharkey M, Condliffe R, Uthoff JM, Alabed S, Metherall P et al Pulmonary hypertension in association with lung disease: quantitative CT and artificial intelligence to the rescue? State-of-the-art review. Diagnostics 2021;11:679. 33918838 10.3390/diagnostics11040679 PMC8070579

[3] Hoeper MM, Lee SH, Voswinckel R, Palazzini M, Jais X, Marinelli A et al Complications of right heart catheterization procedures in patients with pulmonary hypertension in experienced centers. J Am Coll Cardiol 2006;48:2546–52. 17174196 10.1016/j.jacc.2006.07.061

[4] Hudson ER, Smith TP, McDermott VG, Newman GE, Suhocki PV, Payne CS et al Pulmonary angiography performed with iopamidol: complications in 1,434 patients. Radiology 1996;198:61–5. 8539407 10.1148/radiology.198.1.8539407

[5] Horvat D, Orzan RI, Agoston-Coldea L. A non-invasive approach to pulmonary hypertension. J Clin Med 2025;14:1473–. 40094931 10.3390/jcm14051473 PMC11900574

[6] Goh ZM, Johns CS, Julius T, Barnes S, Dwivedi K, Elliot C et al Unenhanced computed tomography as a diagnostic tool in suspected pulmonary hypertension: a retrospective cross-sectional pilot study. Wellcome Open Res 2024;6:249. 39113847 10.12688/wellcomeopenres.16853.2 PMC11303945

[7] Ng CS, Wells AU, Padley SP. A CT sign of chronic pulmonary arterial hypertension: the ratio of main pulmonary artery to aortic diameter. J Thorac Imaging 1999;14:270–8. 10524808 10.1097/00005382-199910000-00007

[8] Chen C, Qin C, Qiu H, Tarroni G, Duan J, Bai W et al Deep learning for cardiac image segmentation: a review. Front Cardiovasc Med 2020;7:25. 32195270 10.3389/fcvm.2020.00025 PMC7066212

[9] Alnasser TN, Hokmabadi A, Sharkey MJ, Maiter A, Dwivedi K, Salehi M et al Advancements in cardiac structures segmentation: a comprehensive systematic review of deep learning in CT imaging. Front Cardiovasc Med 2024;11:1323461. 38317865 10.3389/fcvm.2024.1323461 PMC10839106

[10] Sharkey M, Taylor JC, Alabed S, Dwivedi K, Kavita K, Johns C et al Fully automatic cardiac four chamber and great vessel segmentation on CT pulmonary angiography using deep learning. Front Cardiovasc Med 2022;9:983859. 36225963 10.3389/fcvm.2022.983859 PMC9549370

[11] Jacob AJ, Abdelkarim O, Zook S, Kragholm KH, Gupta P, Cocker M et al AI-based, automated chamber volumetry from gated, non-contrast CT. J Cardiovasc Comput Tomogr 2023;17:336–40. 37612232 10.1016/j.jcct.2023.08.001

[12] Juergens KU, Seifarth H, Range F, Wienbeck S, Wenker M, Heindel W et al Automated threshold-based 3D segmentation versus short-axis planimetry for assessment of global left ventricular function with dual-source MDCT. AJR Am J Roentgenol 2008;190:308–14. 18212214 10.2214/AJR.07.2283

[13] Kirişli HA, Schaap M, Klein S, Papadopoulou SL, Bonardi M, Chen CH et al Evaluation of a multi-atlas based method for segmentation of cardiac CTA data: a large-scale, multicenter, and multivendor study. Med Phys 2010;37:6279–91. 21302784 10.1118/1.3512795

[14] Hurdman J, Condliffe R, Elliot CA, Davies C, Hill C, Wild JM et al ASPIRE registry: assessing the spectrum of pulmonary hypertension identified at a REferral centre. Eur Respir J 2011;39:945–55. 21885399 10.1183/09031936.00078411

[15] Fedorov A, Beichel R, Kalpathy-Cramer J, Finet J, Fillion-Robin JC, Pujol S et al 3D Slicer as an image computing platform for the Quantitative Imaging Network. Magn Reson Imaging 2012;30:1323–41. 22770690 10.1016/j.mri.2012.05.001 PMC3466397

[16] Alnasser TN, Hokmabadi A, Sharkey MJ, Maiter A, Dwivedi K, Salehi M et al A fully automated explainable predictive model for diagnosing pre-capillary and post-capillary pulmonary hypertension on routine unenhanced CT: results from the ASPIRE registry. Euro Heart J—Digital Health 2025;7:ztaf124. 10.1093/ehjdh/ztaf124 PMC12821070 41574043

[17] Isensee F, Jaeger PF, Kohl SAA, Petersen J, Maier-Hein KH. nnU-Net: a self-configuring method for deep learning-based biomedical image segmentation. Nat Methods 2020;18:203–11. 33288961 10.1038/s41592-020-01008-z

[18] Antiga L, Piccinelli M, Botti L, Ene-Iordache B, Remuzzi A, Steinman DA. An image-based modeling framework for patient-specific computational hemodynamics. Med Biol Eng Comput 2008;46:1097–112. 19002516 10.1007/s11517-008-0420-1

[19] Ronneberger O, Fischer P, Brox T. U-Net: convolutional networks for biomedical image segmentation. Lecture Notes in Computer Science 2015;9351:234–41.

[20] Wasserthal J, Breit HC, Meyer MT, Pradella M, Hinck D, Sauter A et al TotalSegmentator: robust segmentation of 104 anatomic structures in CT images. Radiology 2023;5:e230024. 37795137 10.1148/ryai.230024 PMC10546353

[21] Choi JS, Lee SH, Leem A, Song JH, Chung KS, Jung JY et al Prognostic impact of the ratio of the main pulmonary artery to that of the aorta on chest computed tomography in patients with idiopathic pulmonary fibrosis. BMC Pulm Med 2019;19:81. 30999878 10.1186/s12890-019-0843-5 PMC6472007

[22] Monaghan TF, Rahman SN, Agudelo CW, Wein AJ, Lazar JM, Everaert K et al Foundational statistical principles in medical research: sensitivity, specificity, positive predictive value, and negative predictive value. Medicina (B Aires) 2021;57:503. 10.3390/medicina57050503 PMC8156826 34065637

[23] Safari S, Baratloo A, Elfil M, Negida A. Evidence based emergency medicine part 2: positive and negative predictive values of diagnostic tests. Emergency 2015;3:87. 26495390 PMC4608333

[24] Ghigna MR, Dorfmüller P. Pulmonary vascular disease and pulmonary hypertension. Diagnostic Histopathol 2019;25:304–12.

[25] Nakamura K, Akagi S, Ejiri K, Taya S, Saito Y, Kuroda K et al Pathophysiology of group 3 pulmonary hypertension associated with lung diseases and/or hypoxia. Int J Mol Sci 2025;26:835–. 39859549 10.3390/ijms26020835 PMC11765551

[26] Chen HH, Liu CM, Chang SL, Chang PYC, Chen WS, Pan YM et al Automated extraction of left atrial volumes from two-dimensional computer tomography images using a deep learning technique. Int J Cardiol 2020;316:272–8. 32507394 10.1016/j.ijcard.2020.03.075

[27] Astudillo P, Mortier P, Bosmans J, De Backer O, de Jaegere P, Iannaccone F et al Automatic detection of the aortic annular plane and coronary ostia from multidetector computed tomography. J Interv Cardiol 2020;2020:9843275. 32549802 10.1155/2020/9843275 PMC7275208

[28] Zhao W, Guo J, Dong N, Hei H, Duan X, Shen C. Diagnostic value of 3D volume measurement of central pulmonary artery based on CTPA images in the pulmonary hypertension. BMC Med Imaging 2023;23:211. 38093192 10.1186/s12880-023-01180-6 PMC10720078

