# Supplementary material for: AI-based pulmonary artery to ascending aorta ratio on non-contrast CT for pulmonary hypertension: diameter vs. volume assessment
Source: Eur Heart J Imaging Methods Pract. 2026 Aug 24;4(3):qyag146. doi: 10.1093/ehjimp/qyag146 (PMC13543682; doi:10.1093/ehjimp/qyag146)
Supplement: Supplementary file 1 [file qyag146_supplementary_data.docx]

**Supplementary Tables**

**Table S1.** Descriptive summary of PA, AAo and PA/Ao measurements across diameter-based and volume-based deep learning methods (n = 425).

|  | **Diameter-based (mm)** | **Volume-based (mL)** |
| --- | --- | --- |
| **PA** | 30.4 ± 5.5 | 128.2 ± 43.5 |
| **AAo** | 31.9 ± 4.3 | 121.9 ± 39.6 |
| **PA/AAo** | 0.96 ± 0.2 | 1.10 ± 0.4 |

**Table S2.** Agreement analysis between methods and observers using bias and limits of agreement

| **Comparison** | **Bias (Mean difference)** | **Standard deviation** | **Lower LOA** | **Upper LOA** |
| --- | --- | --- | --- | --- |
| **Diameter-based DL vs Observer 1** | -0.014 | 0.113 | -0.236 | 0.207 |
| **Diameter-based DL vs Observer 2** | -0.018 | 0.109 | -0.232 | 0.196 |
| **Volume-based DL vs Observer 1** | 0.016 | 0.274 | -0.522 | 0.553 |
| **Volume-based DL vs Observer 2** | 0.012 | 0.276 | -0.529 | 0.553 |
| **Observer 1 vs Observer 2**  **(Diameter-based Manual Measurments)** | -0.004 | 0.057 | -0.116 | 0.109 |

**Table S3.** Comparison of the two deep learning approaches and observers for PA/Ao ratio (n=97)

|  | **Observer 1** | **Observer 2** |
| --- | --- | --- |
| **Diameter-based DL** | McNemar p = 0.383 / DeLong p = 0.012 | McNemar p = 0.035 / DeLong p = 0. 038 |
| **Volume-based DL** | McNemar p = 0.541 / DeLong p = 0. 009 | McNemar p = 0.096 / DeLong p = 0.031 |

**Table S4.** Diagnostic performance of the two deep learning approaches on internal and external cohorts (n=425)

|  | **Diameter-based DL** | | **Volumetric-based DL** | |
| --- | --- | --- | --- | --- |
|  | **Internal (n=385)** | **External (n=40)** * | **Internal (n=385)** | **External (n=40)** * |
| **Sensitivity [CI]** | 42%  [36.3% - 47.1%] | 36%  [22.5% - 52.4%] | 67%  [61.2% - 71.5%] | 64%  [47.6% - 77.5%] |
| **Specificity [CI]** | 84%  [73.3% - 90.7%] | 50%  [15.0% - 85.0%] | 88%  [78.5% - 93.9%] | 100%  [51.0% - 100%] |
| **PPV [CI]** | 92%  [86.8% - 95.7%] | 87%  [62.1% - 96.3%] | 96%  [93.0% - 98.1%] | 100%  [85.7% - 100%] |
| **NPV [CI]** | 24%  [18.6% - 29.3%] | 8%  [2.2% - 25.0%] | 36%  [29.2% - 43.7%] | 24%  [10.7% - 43.6%] |
| **AUC [CI]** | 0.74  [0.67 - 0.80] | 0.64  [0.31 - 0.96] | 0.86  [0.81 - 0.91] | 0.83  [0.68 - 0.98] |

**Confidence interval width differs due to differences in denominator size.*

**Table S5.** Intraclass correlation with p-values assessing the agreement between diameter-based DL approaches and manual measurements of the PA/AAO ratio.

| **PA/AAo Measurements Correlation** | | **Manual Measurements** | |
| --- | --- | --- | --- |
|  |  | **Observer 1** | **Observer 2** |
| **Diameter-based DL** | **Approach 1** | ICC= 0.79 [0.70 – 0.85]  p < 0.001 | ICC= 0.79 [0.70 – 0.86]  p < 0.001 |
|  | **Approach 2** | ICC= 0.78 [0.69 – 0.85]  p < 0.001 | ICC= 0.79 [0.70 – 0.85]  p < 0.001 |
| **Observer 1 and Observer 2** | | ICC= 0.94 [0.91 – 0.96], p < 0.001 | |

**Table S6.** Diagnostic performance metrics for diameter-based DL approaches and manual diameter measurements compared to right heart catheterisation (RHC).

| **PA/AAo Measurements** | | **RHC (mPAP > 20 mmHg)** | | | | |
| --- | --- | --- | --- | --- | --- | --- |
|  |  | **Sensitivity**  **[CI]** | **Specificity**  **[CI]** | **PPV**  **[CI]** | **NPV**  **[CI]** | **AUC**  **[CI]** |
| **Manual** | **Observer 1** | 57%  [43.3% - 69.0%] | 71%  [53.7% - 85.4%] | 78%  [62.9% - 88.8%] | 48%  [34.0% - 62.4%] | 0.72  [0.62 – 0.83] |
|  | **Observer 2** | 65%  [51.3% - 76.3%] | 74%  [56.7% - 87.5%] | 82%  [68.0% - 91.2%] | 54%  [39.2% - 68.6%] | 0.74  [0.63 – 0.84] |
| **Diameter-based DL** | **Approach 1** | 57%  [43.3% - 69.0%] | 86%  [69.7% - 95.2% | 88%  [73.2% - 95.8%] | 53%  [39.0% - 66.0%] | 0.83  [0.74 – 0.91] |
|  | **Approach 2** | 53%  [39.0% - 65.8%] | 87%  [73.3% - 95.8%] | 86%  [69.7% - 95.2%] | 56%  [43.3% - 68.8%] | 0.81  [0.72 – 0.90] |
